# Mature Teratoma at the Lumbar Spinal Cord: A Case Report and Literature Review

**DOI:** 10.7759/cureus.52307

**Published:** 2024-01-15

**Authors:** Lilian Zavala-Romero, Eliezer Villanueva-Castro, Rudradeep Datta-Banik, Alexis Genaro Ortiz-Altamirano, María Magdalena Rodriguez-Esquivel, Jesús Cienfuegos-Meza, Juan Nicasio Arriada-Mendicoa

**Affiliations:** 1 Department of Neurosurgery, Instituto Nacional de Neurología y Neurocirugía Manuel Velasco Suárez, Mexico City, MEX; 2 Department of Neuropathology, Instituto Nacional de Neurología y Neurocirugía Manuel Velasco Suárez, Mexico City, MEX

**Keywords:** lumbar intradural tumor surgery, teratoma, intramedullary tumor, neurosurgery, spinal cord tumor surgery, spinal teratoma

## Abstract

Mature spinal teratoma is a rare type of germ cell tumor that arises from any of the three germ cell layers (ectoderm, mesoderm, and endoderm) and consists of differentiated tissues and structures that reflect the cellular organization and morphology of normal adult tissues. It has the ability to grow independently and cause compressive symptoms when found in this rare location. In this article, we present the case of a 29-year-old male patient with the onset of neurological symptoms beginning with pelvic limb paresthesias and progressing to back pain. Magnetic resonance imaging (MRI) revealed a tumor at L1-L4, which was resected by laminotomy, and histopathology revealed a mature intradural teratoma. Fortunately, this histologic type had a good prognosis for our patient, who had a significant clinical improvement.

A systematic review was performed using the Preferred Reporting Items for Systematic Reviews and Meta-Analyses (PRISMA) methodology with PubMed and Google Scholar to find similar case reports and to summarize the main features of this disease, which contributes to the understanding of its diagnostic presentation, treatment, and prognosis, improving clinical practice in the management of similar cases. The rarity of this condition, together with its wide clinical heterogeneity and prognosis, underscores the importance of a thorough evaluation of cases of intramedullary lesions, where the consideration of uncommon diseases in the differential diagnosis should be highlighted.

## Introduction

Teratomas are a type of pluripotent cell tumor derived from the three germ cell layers [1]. They are not commonly encountered in the central nervous system, accounting for only 2% of cases, with an incidence of 0.15% to 0.18% among spinal tumors. Histologically, they can be classified into three subtypes: mature teratoma, immature teratoma, and teratoma with malignant transformation [2]. The first subtype is associated with a favorable prognosis, boasting a 10-year survival rate of over 90% [3]. Treatment for these cases typically revolves around complete surgical resection, as chemotherapy and radiosurgery yield minimal effects [4]. Our aim is to present a case involving an adult diagnosed with lumbar teratoma, an uncommon spinal tumor. The rarity of its occurrence in this age group, coupled with its specific location and presentation, prompts us to delve into the main characteristics associated with this type of pathology.

## Case presentation

We present the case of a 29-year-old patient with a complex history and a challenging diagnostic and therapeutic journey. The symptoms began at the age of 11 years with paresthesias in the phalanges of the feet, which resolved spontaneously or with the use of medication. Over the years his symptoms progressed to low back pain with radiation and hypoesthesia in the left lower limb, together with recurrent episodes of intense paresthesias in the popliteal fossa (aggravated by physical activity), dizziness, tinnitus and abnormalities in gait and proprioception. The patient reported that he occasionally couldn't "feel" his left lower limb, which was the cause of subsequent falls. Six months before the first medical consultation, constipation and left lower extremity weakness were added. He underwent a series of magnetic resonance imaging (MRI) scans at a community hospital, which revealed an intradural lesion extending from L1 to L4 (lumbar spinal level). The patient was referred to the "Manuel Velasco Suárez" National Institute of Neurology and Neurosurgery, as the lesion was suspected to be of oncological origin. The delay in diagnosis from symptom onset was due to late referral because the patient resided in a rural area with limited access to medical services.

**Figure 1:**
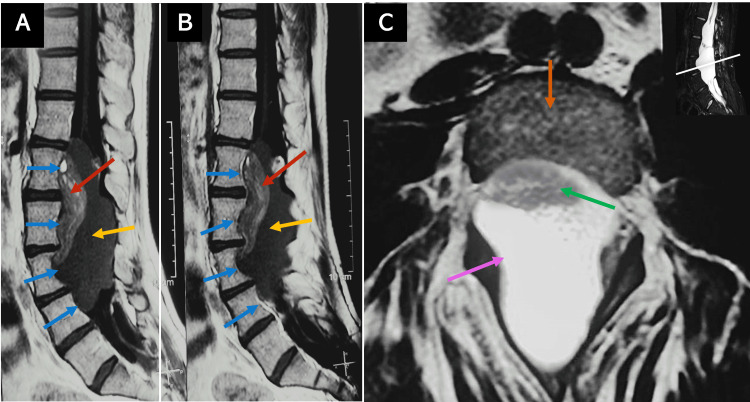
Preoperative MRI (A, B) Preoperative sagittal T1 MRI of the lumbar spine showing a heterogeneous lumbar tumor lesion with well-defined borders that modifies the morphology of the L1-L4 vertebral bodies (blue arrows). It presents a ventral hyperintense component, a head tumor with fat tissue (red arrow), and a dorsal hypointense component, a body tumor (yellow arrow). (C) Axial T2 MRI at L3 shows the intradural body component of the tumor occupying the entire diameter of the spinal canal (pink arrow). The head of the teratoma is indicated by the green arrow. The vertebral body corresponds to the orange arrow. MRI: Magnetic resonance imaging, L: lumbar spinal level

On admission to our hospital, a neurological examination was performed which showed intact cranial nerves, reduced muscle strength in both lower limbs (3/5 score on the Daniels scale), hyperreflexia (+++) in the left lower limb, together with paresthesias in the left foot, reduced vibratory sensation in the left lower limb (at L1-L4 dermatome levels), and localized pain in the paravertebral and left iliotibial band regions. The neurological examination was otherwise normal and sphincter control was not impaired. The anal tonus was normal and urinary incontinence was not present. Routine laboratory examinations were in the normal range. Somatosensory evoked potentials revealed severe bilateral dysfunction of the proprioceptive pathways of both lower limbs, resulting in an absolute conduction block at the level of the thoracolumbar segment of the gracilis fasciculus. On examination, there was no gross evidence of cutaneous abnormalities, dermal sinus tracts, or a history of spinal dysraphism. No palpable midline spinal defects were appreciated. He denied any previous lumbar surgeries or lumbar puncture. Imaging evaluation showed a tumor with well-defined borders, heterogeneous composition, with a ventral hyperintense zone that modified the morphology of the L1-L4 vertebral bodies (Figure 1). Contrasted imaging studies could not be performed because the patient was allergic to gadolinium.

**Figure 2:**
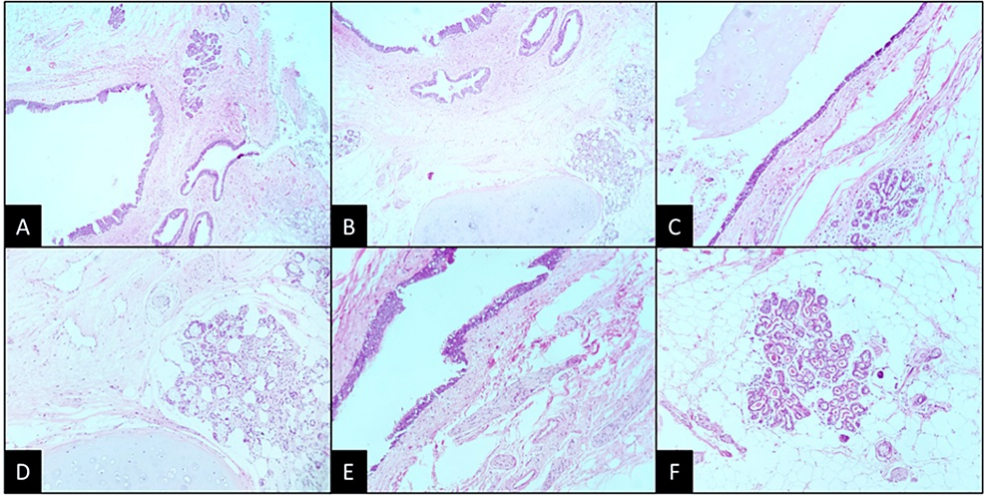
Histopathological findings (A) Panoramic view of a cyst delineated by stratified epithelium. The cystic wall is composed of tissue derived from the three germinal cell layers (H&E; 4x). (B) Presence of mature cartilage, glands, and stratified epithelium that invaginates and forms dilated tubules (H&E; 4x). (C) Well-formed acini are immersed in adipose tissue (H&E; 10x). (D) Structures similar to Paccinian corpuscules are shown (H&E; 10x). (E) The epithelium is columnar, pseudostratified with cilia, and goblet cells. Some nervous tracts can be seen (H&E; 10x). (F) Acini of apocrine glands within mature adipose tissue (H&E; 10x). H&E: hematoxylin and eosin

Based on the clinical presentation and imaging findings, a preliminary diagnosis of probable medullary meningioma at the L1-L4 levels of the spine was made. The patient underwent fluoroscopy-guided surgery with intraoperative neuromonitoring in January 2017; the procedure consisted of bilateral L2-L4 laminotomy followed by microsurgical resection of the intradural lesion and laminoplasty. Intraoperatively, a firm, yellowish nodule measuring 1.2 x 1.1 x 0.7 cm was identified as adherent to bony fragments. Gross excision of the intramedullary lesion was achieved, including total removal of the tumor capsule. Dissection of the solid mass component revealed its extension into the intramedullary region. The lumbar lesion was sent for neuropathological examination which revealed a mature teratoma as the final diagnosis (Figure 2). The observed tissues comprised mature squamous epithelium, adnexal glandular tissue, adipose tissue, peripheral nerve tissue, and glial tissue. Furthermore, no immature elements were identified.

**Figure 3:**
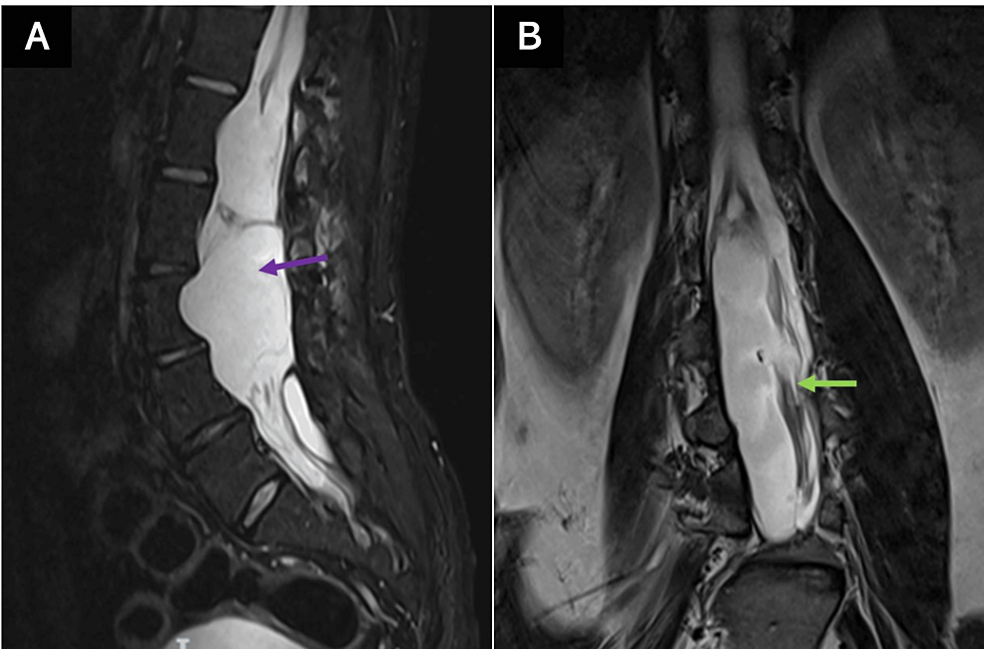
Postoperative MRI (A) Postoperative sagittal T2 MRI without residual tumor, with occupation of cerebrospinal fluid in the site where the tumor was located (purple arrow). (B) Postoperative sagittal T2 MRI cauda equina displacement (green arrow). MRI: magnetic resonance imaging

The clinical follow-up was carried out every three months during the first year and every six months after the second year of surgery. Likewise, radiological controls of the lesion were carried out. Postoperative MRI determined total resection of the tumor mass, with the presence of cerebrospinal fluid at the site of the teratoma, as well as displacement of the cauda equina (Figure 3).

The patient underwent physical rehabilitation therapy and progressively his clinical condition enhanced. In the most recent physical examination, the patient had a strength of 4/5 on the Daniels scale in both lower extremities, which the patient and his caregiver referred to as a substantial improvement. Cranial nerve assessment and neurological examination of the rest of the body segments remained without deficits. Although proprioception has improved, he continues to have some deficits in gait coordination. Similarly, paresthesias partially disappeared. Currently, five years after surgery, the patient has a functional state that allows him to perform most of his activities.

Materials and methods

**Figure 4:**
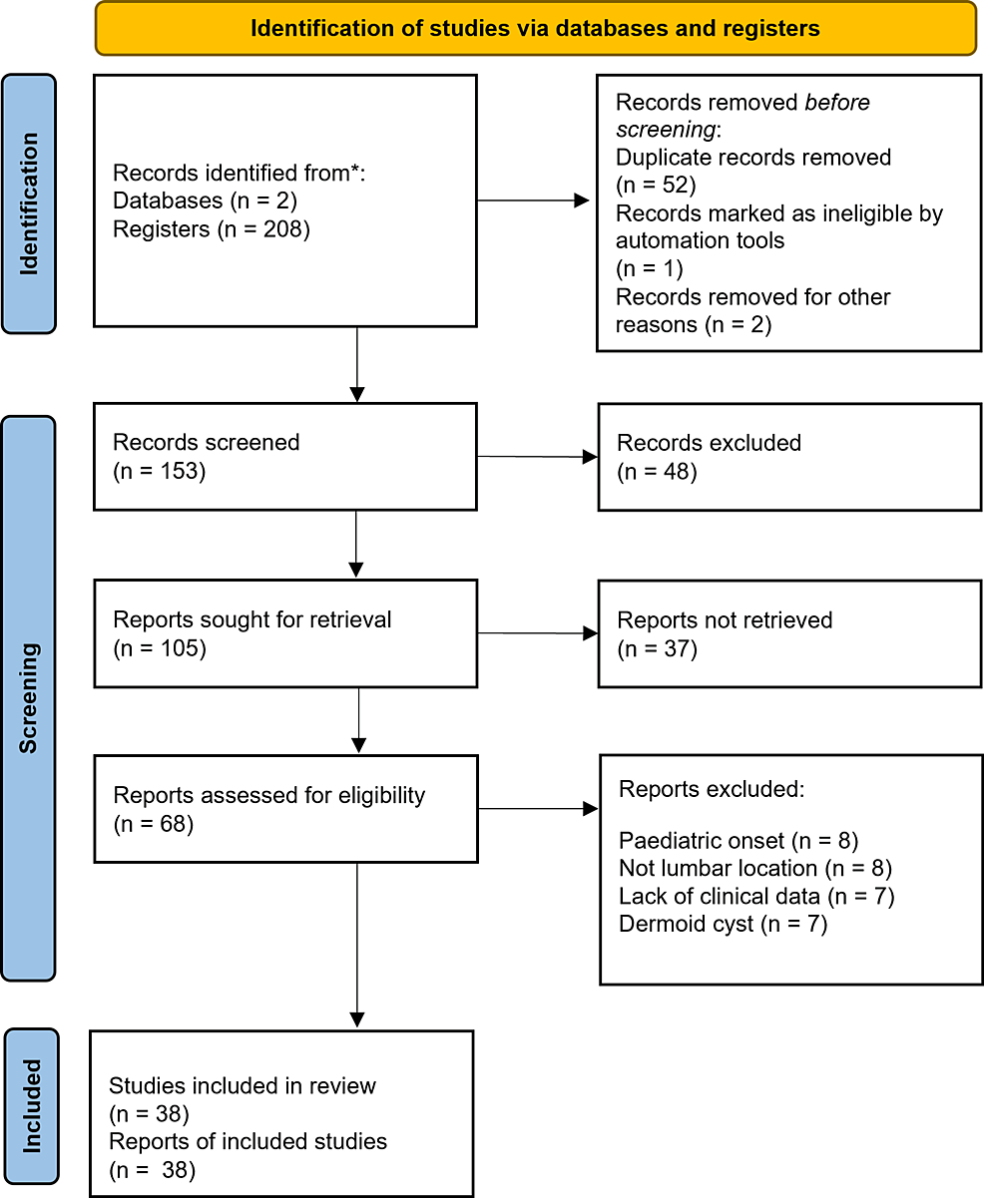
PRISMA flow chart Preferred Reporting Items for Systematic Reviews and Meta-Analyses (PRISMA) flow chart [5].

We performed a systematic review using the PRISMA (Preferred Reporting Items for Systematic Reviews and Meta-Analyses) guide [5] of intramedullary lumbar tumors with the histopathological diagnosis of mature teratoma. Our eligibility criteria were included if they primarily or in part show information on a case report or case series of intramedullary lumbar teratoma in adults. We excluded articles whose full text is not in English, and articles not found. Search strategy In the PubMed search engine consisted of the item (teratoma) AND ((spinal cord) OR (spinal)) AND (adult) AND (intramedullary). The database coverage was established from 1966 to 2023. In the Google Scholar search engine, the search was done by requesting articles with the terms “spinal cord”, “teratoma”, “mature”, “adult”, “intramedullary”, “lumbar”, somewhere in the report. Primarily 69 results were obtained from the PubMed search and 141 results from Google Scholar. Repeated results within the searches were later eliminated (52 records). The results were arranged by relevance according to the search engine. The abstract of the results in both searches was read by four reviewers independently. The abstracts were carefully read by reading the title and abstract. Cases in which no information could be gathered (because of language barriers or missing data) were excluded. Extramedullary, immature, cervical, or sacrococcygeal teratomas, dermoid cysts, and pediatric-onset teratomas were excluded from the analyses. All articles were evaluated to assess relevance for this review. After the initial screening, they carefully read the complete abstracts and assessed the entire articles, considering only the most relevant according to their suitability and appropriateness for this review. Articles selected by all five researchers independently in consensus were directly included in the primary analysis. Subsequently, the investigators reached a final agreement on the selected articles to write a discussion focusing on the epidemiological, clinical, diagnostic, surgical, and prognostic aspects of this pathology (Figure 4) [1-4,6-39].

Likewise, a table was made with the reports and series of cases of teratomas (a total of 16 articles and 18 patients) that met these characteristics and that were published in the present century (Table 1).

**Table 1:** Review of adult intradural mature teratoma cases with lumbar location (2000-2023) T: thoracic spinal level, L: lumbar spinal level

Autor	Year	Number of cases	Age/sex	Onset	Location	Associated abnormality	Treatment	Improvement
Acharya Ashish et al. [6]	2020	1	25/male	Lower limbs muscle weakness.	L1	No	Total resection.	Remission of weakness and urinary disturbance.
De Oliveira José Manuel et al. [7]	2019	1	35/female	Lower limb weakness and urinary retention.	L2-L4	No	Partial resection.	Improvement of weakness.
Asan Ziya et al. [8]	2017	1	29/female	Moderate and progressive lower back pain.	T12-L1	No	Total resection.	Remission of pain.
Mohammadi Alireza et al. [9]	2017	1	18/male	Long-term history of lower back pain.	L2-L3	No	Partial resection.	Remission of pain.
Schmidt Richard et al. [10]	2016	1	49/male	Overflow urinary incontinence.	T12-L3	No	Partial resection	Mild improvements in bladder sensation. No pain or weakness.
Turan Nefize et al. [11]	2016	1	48/male	Fasciculations in the bilateral upper and lower extremities.	L2-L3	No	Total resection.	Complete resolution of fasciculations.
Li Yuan et al. [12]	2013	1	22/female	Intermittent pinching pain in the lower right shank.	T12-L2	No	Total resection.	Remission of pain.
Jian Wu et al. [13]	2009	2	Case 1: 18/male; Case 2: 57/male	Case 1: severe and acute low back pain. Case 2: progressive back pain.	Case 1: L2-L4; Case 2: L1-L2	No	Total resection in both cases.	Case 1: Remission of symptoms. Case 2: low weakness in the lower extremities.
Arvin Babak et al. [14]	2009	1	52/female	Lower back pain and muscle weakness.	L2-L5	No	Subtotal resection.	Partial improvement of weakness.
Oh Jae Sang et al. [15]	2009	1	44/male	Voiding difficulty. Spontaneous rupture.	L3-L5	No	Subtotal resection.	Voiding difficulty improved gradually.
Mohindra Sandeep et al. [16]	2008	1	35/male	Intermittent urinary retention and persistent bowel problems (then renal failure).	L1-L5	No	Total resection.	Remission of renal failure.
Kahilogullari Gokmen et al. [17]	2006	1	42/female	Muscle weakness.	L1	No	Total resection.	Remission of symptoms.
Mut Melike et al. [18]	2006	1	34/female	Right lower extremity weakness, left lower extremity sensory deficit, and urinary retention.	L1-L2	Yes (diplomyelia)	Total resection.	Strength returned to normal, neuropathic pain remains present.
Fernández-Cornejo Víctor et al. [19]	2004	1	42/male	Cauda equina syndrome.	L1-L2	No	Total resection.	Remission of symptoms.
Hejazi Nedal and Witzmann Alfred [20]	2003	2	Case 1: 20/male; Case 2: 45/female	Case 1: lumbago and dysesthesia. Case 2: lumbago, then paraparesis.	Case 1: L2-L4; Case 2: T11-L2	No	Total resection in both cases.	Case 1: superficial hypesthesia. Case 2: improve, slight urinary incontinence.
Arai Yasuhisha et al. [21]	2000	1	54/female	Gait disturbance.	L3-L4	Yes (spina bifida)	Total resection.	Satisfied, sensory and weakness improved.

## Discussion

Our case report holds significance because it underscores the importance of considering all diagnostic hypotheses for intramedullary lesions, even the rarest ones such as teratomas. This consideration can significantly impact the surgical approach. The challenging nature of the differential diagnosis lies in the nonspecific clinical characteristics, particularly when associated spinal malformations are absent. This case highlights the complexity of diagnosing and managing rare spinal cord tumors. The presentation of diverse symptoms, the need for interdisciplinary collaboration, and the patient's response to surgical and medical interventions underscore the challenges posed by such cases.

Epidemiology and classification

Teratomas are tumors that can arise from any of the three germ cell layers - ectoderm, mesoderm, and endoderm - and they have the ability to grow on their own [22]. According to the 2021 WHO Central Nervous System (CNS) classification, these tumors are classified as germ cell tumors and can be categorized into three histological types: mature teratoma, immature teratoma, and teratoma with somatic-like neoplasia [23]. A mature teratoma is characterized by differentiated tissues and structures in its composition that reflect the cellular organization and morphology of normal adult tissues. This review will focus on this type of variant. In contrast, immature teratomas account for approximately 10-50% of all teratomas but are rare entities in the central nervous system [24]. The histopathologic diagnosis of this variant of teratoma is made when incompletely differentiated components are documented in tissues of fetal or embryonic origin. Some of these suggestive findings include hypercellular embryonic mesenchyme, primitive neuroectodermal elements, and clefts lined by melanotic neuroepithelium. The biological behavior of these tumors is variable, and they have an intermediate prognosis between mature teratomas and high-risk germ cell tumors [1,25]. Similarly, finding immature teratoma on biopsy has been significantly associated with an increased risk of developing growing teratoma syndrome (GTS) [26,27].

Teratomas are included in a group of tumors classified as inclusion tumors. Classically, teratomas are thought to originate from aberrant pluripotential cells of the yolk sac migrating to the amnion during embryogenesis. Teratomas in the CNS are very rare, accounting for only 2% of all teratomas [2]. They are usually located inside the skull, especially near midline structures. The pineal region is the most common location, followed by the suprasellar compartment. Although they can also occur in other areas of the brain, such as the posterior fossa, fourth ventricle, cerebral hemispheres, and basal ganglia. Spinal cord teratomas are exceptionally rare, accounting for only 0.1-0.5% of all spinal cord tumors [19,28]. In a study of 1322 spinal cord tumors, only two cases were identified as teratomas [19]. Hazneci et al. reported that 66% of spinal teratomas occur in the lumbar region, and 57% of them are intramedullary [28]. Teratomas are more frequently associated with spinal malformations in children than in adults [19,20]. According to Prasad et al., teratomas typically occur in men aged between 40 and 50 years [22]. In our case, we suspect that the patient belongs to this group since he began experiencing symptoms many years before coming to our institute.

Clinical manifestations

There is a vast variety of clinical manifestations of spinal cord teratomas in adults. Typically, the presentation is influenced by factors such as the tumor location (cervical, thoracic, or lumbar spine), as well as the level of infiltration (intramedullary, intradural extramedullary, or extradural). In a case series by Poeze et al. [29], which reviewed 31 cases of intramedullary teratomas, the most common symptom was motor dysfunction, observed in 22 cases (71%), followed by abnormal reflexes (16 cases, 52%) and sensory alterations (14 cases, 45%). Urinary problems and pain were reported in 11 cases (35%). Other less common presentations, observed in only one or two patients, included meningismus, defecation abnormalities, sexual dysfunction, and visible tumors. Interestingly, the histological subtype did not appear to predict the presenting symptoms. Among the reported symptoms, the predominant presenting feature was sensory disturbances. Additionally, it displayed a classical clinical presentation involving the posterior cords, marked by proprioceptive ataxia - a symptom infrequently associated with these types of tumors. According to Prasad et al. (2020), the most common symptoms include pain, progressive limb weakness, and bowel/bladder disturbances. An acute onset presentation has been rarely described in earlier reports [22].

Moon H et al. (2010) describe that abrupt shifts in symptom patterns might manifest after minor injuries. Nonetheless, a sudden onset or alteration in symptoms should raise suspicion for an immature or malignant teratoma, although these are much rarer, they tend to progress more aggressively [30].

Diagnosis

Published information regarding the diagnosis and prognosis of adult-onset intradural spinal teratoma is limited due to its rarity. Most reports describe isolated cases or fewer than three cases in a series. It is often challenging to distinguish teratomas from other spinal cord tumors in imaging studies. When X-rays are used, some nonspecific findings like the erosion of vertebral bodies and elongation of the intervertebral spaces can be observed [2]. Computed tomography (CT) scans may sometimes reveal differences in densities within the tumor, suggesting the presence of different tissue types in the tumor, and may occasionally show calcification or ossification within the tumor or in the covering membranes, but these findings lack specificity [31]. MRI is considered as the most valuable imaging modality for the detection of these tumors. In the MRI scans, spinal teratomas often exhibit a lobulated or cystic appearance, with signal intensity and enhancement patterns that vary based on the solid and cystic components of the tumor. They frequently present as hyperintense lesions on T1-weighted sequences, attributable to their fatty composition [28]. Generally, the primary role of MRI is in surgical planning, aiding in the localization of the lesion, determination of whether it is intra- or extradural and/or intramedullary, and assessment of the likelihood of containing a sizable cystic component [32]. In our experience, spinal teratomas exhibit slow progression during the initial stages; the primary diagnostic tool of significance is MR imaging. In instances where the tumor causes damage to the bony structures, radiography or CT imaging could prove beneficial for diagnosis due to the indirect signs they present.

Intramedullary spinal teratomas have infrequently been associated with split cord malformations, such as diastematomyelia and diplomyelia, which are important factors to consider when planning surgery and assessing the patient's prognosis [18]. Spinal teratomas emerging concomitant with spinal dysraphic lesions have been presumed to have a dysembryonic origin as other tumors (such as medulloblastoma) that occur in the midline [33]. This theory, the most widely accepted, postulates that teratomas develop through the differentiation of pluripotent cells within the primitive streak or cell mass [34].

The initial diagnostic approach for these tumors involves a combination of clinical and radiological findings. This is followed by histopathological analysis, where a diagnosis is made based on findings like the presence of tissues of endomesoectodermal origin with undifferentiated germ layer cells, as it was observed in our case where the histopathological study reported mature cartilage, glands, and keratinized epithelium with tubules, adipose tissue, and Paccini's corpuscle-like tissue. Similarly, other case studies report histopathological findings such as numerous fatty cysts consisting of neuroepithelial and epithelial tissues, nerve cells, specialized structures resembling Pacini's corpuscles, ganglion cells and Rosenthal cells, cutaneous elements, focal calcifications, thyroid follicles, and carcinoid features. Thus from this, it is inferable that a teratoma diagnosis should be suspected whenever structures derived from one or more germ layers are present in the biopsy [12,28,35,36]. Some immunohistochemical markers could improve diagnostic accuracy, these include mainly SALL4, AFP, and b-HCG reported by Mukasa in 2017 [36].

Treatment

Surgical removal is the mainstay of treatment for teratomas, where a complete gross resection should be aimed [37], as there is a risk of regrowth of the residual endodermal and ectodermal tissue from the lesion. Nevertheless, Allsopp et al. also reported that complete resection vs macroscopic resection has similar recurrence rates, 9% and 11% respectively [36], and the time period of regrowth ranges from 3 to 13 years [38], in addition, there is no clear evidence of adjuvant therapies for these tumors [35]. When performing tumor resections at the cauda equina level, various surgical approaches are available. Some authors opt for laminotomy to excise the tumor while safeguarding the integrity of posterior anatomical structures. This technique offers the benefit of straightforward postoperative MRI monitoring. Long-term follow-up is essential, monitoring symptoms, neurological alterations, and the possibility of spinal deformities [6,8].

Patients with mature teratomas usually have a good prognosis as this histologic variety has been reported to have a 10-year survival rate of 92% [3]. The examination of the quality of life and psychological aspects linked to the initial appearance and treatment of spinal teratomas has not been specifically explored. Nevertheless, evaluations of extramedullary intradural tumors have indicated that sphincter dysfunction, sensorimotor issues, and motor deficits profoundly affected the quality of life, with sphincter dysfunction showing the most pronounced impact [39].

## Conclusions

Teratomas of the spinal cord in adults, mainly intramedullary, are exceptionally rare entities; there are different histological varieties, however, the mature teratoma is the most frequent. These neoplasms present various symptoms mainly due to compression and are usually diagnosed by MRI and histopathological study. Treatment strategies depend on the location of the tumor and the degree of neurological involvement since it should be considered that there are emerging teratomas concomitant with spinal dysraphism. The most commonly used treatment is total gross resection; the use of adjuvant therapies is not well established yet.
